# The Presence of the Iron-Sulfur Motif Is Important for the Conformational Stability of the Antiviral Protein, Viperin

**DOI:** 10.1371/journal.pone.0031797

**Published:** 2012-02-21

**Authors:** Shubhasis Haldar, Simantasarani Paul, Nidhi Joshi, Anindya Dasgupta, Krishnananda Chattopadhyay

**Affiliations:** 1 Structural Biology and Bioinformatics, Indian Institute of Chemical Biology, Council for Scientific and Industrial Research, Kolkata, West Bengal, India; 2 Infectious Diseases and Immunology Divisions, Indian Institute of Chemical Biology, Council for Scientific and Industrial Research, Kolkata, West Bengal, India; Koç University, Turkey

## Abstract

Viperin, an antiviral protein, has been shown to contain a CX_3_CX_2_C motif, which is conserved in the radical S-adenosyl-methionine (SAM) enzyme family. A triple mutant which replaces these three cysteines with alanines has been shown to have severe deficiency in antiviral activity. Since the crystal structure of Viperin is not available, we have used a combination of computational methods including multi-template homology modeling and molecular dynamics simulation to develop a low-resolution predicted structure. The results show that Viperin is an α -β protein containing iron-sulfur cluster at the center pocket. The calculations suggest that the removal of iron-sulfur cluster would lead to collapse of the protein tertiary structure. To verify these predictions, we have prepared, expressed and purified four mutant proteins. In three mutants individual cysteine residues were replaced by alanine residues while in the fourth all the cysteines were replaced by alanines. Conformational analyses using circular dichroism and steady state fluorescence spectroscopy indicate that the mutant proteins are partially unfolded, conformationally unstable and aggregation prone. The lack of conformational stability of the mutant proteins may have direct relevance to the absence of their antiviral activity.

## Introduction

The role of antiviral genes in hosts has been the subject of intense research over the last decade. Many of the antiviral genes present in the hosts are normally silent, but get induced in response to viral infection predominantly via the interferon mediated pathways and are known as Interferon Stimulated Genes (ISG). More than 50 years ago, the interferon system was discovered as an antiviral defense mechanism. Since then several genes have been implicated in anti-viral responses. One of the better studied IFN inducible anti-viral genes is Viperin (virus inhibitory protein, endoplasmic reticulum-associated, interferon-inducible) which has been shown to inhibit virus replication using a variety of model systems including Cytomegalovirus (CMV) [1], Influenza Virus [2], Vesicular Stomatitis Virus [3] and Hepatitis C Virus [4]. Viperin was originally identified as a CMV induced gene by Shenk and colleagues and was named *cig5* (Cytomegalovirus induced gene 5). It was later shown by Cresswell and colleagues that cig5 is also induced by both Type I and Type II Interferons and when pre-expressed in cells block the production of infectious virus particles [1]. The same group showed in a subsequent study that Viperin blocks budding of Influenza virus particles at the plasma membrane [2]. While virus entry, gene expression and protein synthesis were unchanged, Viperin caused virus particles to prevent budding out of the cell thereby creating a “stalk like” or “daisy chain” structure where virus particles are trapped at the plasma membrane and cannot bud out. It was also shown that Viperin interacts with the lipid metabolism enzyme farnesyldiphosphate synthase (FPPS) thereby disrupting lipid rafts, which Influenza uses for budding. Knockdown of FPPS by siRNA resulted in the abrogation of the ability of Viperin to block virus budding [2].

**Figure 1:**
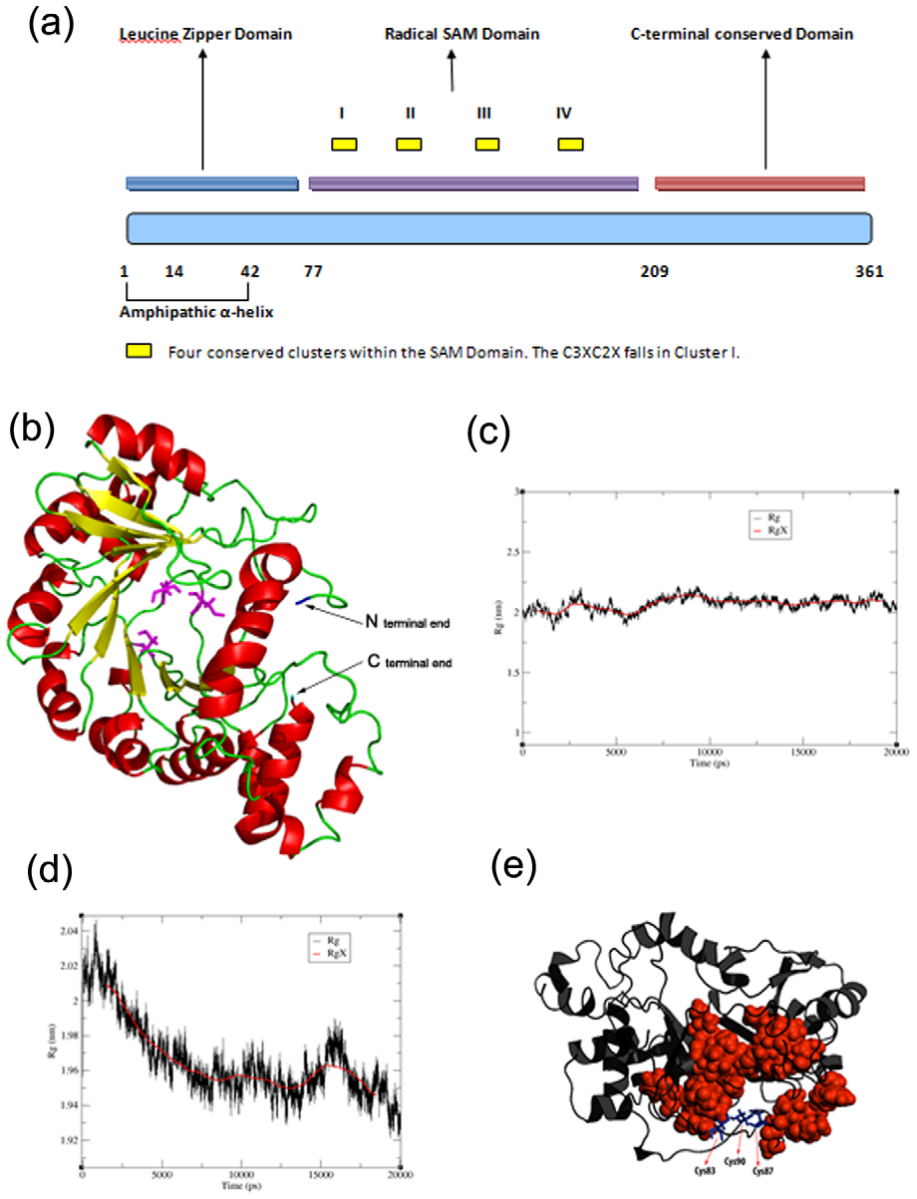
Viperin domain representation, predicted model, structural property and hydrophobic environment of Viperin surrounding three cysteines. (a) The domain representation of Viperin containing N-terminal amphipathic helix (residues 1–42) shown in blue color rectangular box; a middle domain that contains a conserved S-Adenosyl-L-Methionine (SAM) domain shown in violet color rectangular box and a Leucine Zipper domain; and a highly conserved C terminal domain shown in red color rectangular box. (b) The modeled structure of Viperin. Three cysteines at the positions 83, 87, 90 (shown as sticks of magenta color) are present at the bottom of the central pocket. This pocket has been surrounded by parallel beta strands forming the first layer (shown in yellow color). A layer of alpha helices (shown in red color) is also found just above the beta sheets. All these alpha helices and beta sheets are arranged in alternating order in the primary sequence separated by coils and loops shown in green color. (c) The fluctuations of the radius of gyration (R_g_) of a 20 ns simulation run of WT Viperin. (d) The fluctuations of the radius of gyration (R_g_) of a 20 ns simulation run of triple cysteine mutant of Viperin. (e) Hydrophobic environment of Viperin around three cysteine molecules. Cysteine residues at the positions 83, 87, 90 are shown as sticks of blue color and hydrophobic patches are shown as spheres of red color. All other regions of the protein are shown as black. Cys83 and Cys87 are surrounded by most of the hydrophobic groups whereas Cys90 is placed towards the surface of the protein.

While many of the ISGs have been shown to block viral life cycles at different stages, not much is known about the molecular mechanisms by which they work. Viperin is a highly conserved across mammals as well as lower vertebrates in terms of amino acid sequence. The protein by itself seems to contain 3 distinct domains (a representation of the domain structure is shown in Figure 1(a)). A N-terminal amphipathic helix (residues 1–42), a middle domain that contains a conserved S-Adenosyl-L-Methionine (SAM) domain and a Leucine Zipper domain and a highly conserved C terminal domain. The N terminal end of the protein containing the amphipathic helix has been shown to be necessary for the correct localization and function of the protein [5], [6]. Though the presence of the domain was known earlier, it has only been very recently shown that Viperin is actually an S-Adenosyl-L-methionine (SAM) enzyme [7]. Interestingly, it also possesses a CX_3_CX_2_C motif, as well as some additional motifs found in other radical SAM enzymes [8]. The CX_3_CX_2_C motif is involved in iron-sulfur (Fe-S) cluster binding in several proteins [9]. To examine the role of this motif, Jiang et al made a mutant Viperin harboring the triple mutant where C83, C87 and C90 were all mutated to Alanine. The mutant failed to exhibit anti-viral activity against HCV, showing the importance of the Fe-S binding domain of this protein [4].

In this paper, we have used a number of computational and biophysical techniques to probe the role of the CX_3_CX_2_C motif in the conformation and stability of Viperin. Structure prediction using a multi-template homology modeling complimented by molecular dynamics simulation suggests that Viperin has an intrinsic Fe-S cluster present in the core of the protein. This cluster is surrounded by β-sheets all around and these β-sheets are in turn surrounded by alpha helices. The simulation suggests the probability that the removal of Fe-S cluster from Viperin would lead to the disruption of the overall structure of the protein. To verify these predictions, we have taken a mutational approach to characterize the importance of the C83A, C87A and C90A residues. Using various biochemical and biophysical techniques, we have shown that these cysteine residues are not only important for the binding of iron to the protein, but also to the overall stability of the protein itself. Mutation of even a single cysteine residue results in the unfolding of the protein. Our results also show that the use of 2, 2, 2 trifluoroethanol (TFE) can induce refolding to the mutant proteins, which are natively unfolded. This study thus provides a structure-function basis of the importance of Fe-S binding to Viperin and its possible role in anti-viral function.

## Materials and Methods

Urea and guanidine hydrochloride was purchased from Sigma Chemical Company. 2, 2, 2- trifluroethanol (TFE) was obtained from Fluka Company. All the other reagents used were of the highest purity.

### Cloning and mutagenesis of Viperin

Human Foreskin Fibroblast (HFF) cells were treated with Interferon-α (R&D Systems) at a concentration of 100 ng/ml for 12 hours. Cells were then lysed and RNA extracted using RNeasy kit (Qiagen) using manufacturer's protocol. One step RT-PCR was performed to obtain the cDNA for Viperin using the forward (CGC**GGATCC**ATGTGGGTGCTTACACCTGCT) and reverse (CCC**AAGCTT**CTACTACCAATCCAGCTTCAGATC) primers. The full length cDNA was then digested with Hind III and Bam H1 and cloned into pET-28a vector (Novagen). For site-directed mutagenesis the wild type Viperin construct was used as a template and the following primers were used to generate the single point mutants (C83A, C87A, C90A) and the triplemutant(C83AC87AC90A).C83A:5′CTATCACTTCACTCGCCAG**GCC**AACTACAAATGCGGCTTC3′ and 5′GAAGCCGCATTTGTAGTT**GGC**CTGGCGAGTGAAGTGATAG3′, C87A:5′ CTTCACTCGCCAGTGCAACTACAAA**GCC**GGCTTCTGTTTC3′ and 5′GAAACAG AAGCC**GGC**TTTGTAGTTGCACTGGCGAGTGAAG3′,C90A:5′CAACTACAAATGCGGCTTC**GCT**TTCCACACAGCCAAAAC3′ and 5′GTTTTGGCTGTGTGGAA**AGC**GAAGCCGC ATTTGTAGTTG3′,C83AC87AC90A:5′CTTCACTCGCCAG**GCT**AACTACAAA**GCT**GGCTTC**GCT**TTCCACACAGCC3′ and 5′GGCTGTGTGGAA**AGC**GAAGCC**AGC**TTTGTAGTT **AGC**CTGGCGAGTGAAG3′.

All DNA sequences were confirmed by automated DNA sequencing.

### Expression and Purification of the WT and cysteine mutants

The recombinant proteins were expressed in *Escherichia coli* BL21DE3 strain and were grown to OD_600_ of 0.5. Induction was performed using IPTG (1 mM) for 5 hours at 37°C. Cells were lysed at 15 kpsi pressure by using constant cell disruption system (Constant System Limited, UK). Wild type (WT) protein was expressed in the supernatant while all the mutants were found in the inclusion body. WT Viperin was purified using a Ni^2+^ affinity column under native condition. For the mutant proteins the inclusion body was dissolved in 4 M Guanidinium-HCl and refolded using 20 mM phosphate buffer (pH 7.5) in the presence of FeCl_3_ solution. Refolded proteins were further purified using Ni^2+^ affinity column.

### Molecular dynamics simulations

Molecular dynamics simulations were carried out using GROMACS with OPLS (Optimized Potentials for Liquid Simulations) all atom force field [10], [11] at constant temperature and pressure (NTP) ensemble. The calculations were carried out using an Apple cluster equipped with operating system Darwin (OS release 9.6.0). Eight simultaneous processors were used for parallel processing of mdrun program of GROMACS using Open MPI module (version 1.4.3). The V-rescale coupling was employed to maintain a constant temperature of 308 K with coupling constant of 0.1 ps for both protein and non-protein molecules in the system. Parrinello-Rahman coupling was used to maintain constant semi isotopic pressure of 1 bar with coupling constant of 2 psec within a fixed volume of dodecahedron box (diameter = 0.75 nm) filled with water molecules (spc 216 water model). Periodic boundary condition was employed for defining perfect 3D tiling of the system. The particle mesh Ewald method (PME) with grid spacing of 0.16 nm, was used for electrostatic calculations [12]. A non-bonded cutoff of 1 nm for Lennard-Jones potential was used. The net charge of the protein was compensated by adding 0.1 M NaCl into the system. Steepest descent algorithm with the maximum of five thousand steps was applied for the energy minimization of the protein. Leap-frog integrator was utilized for the molecular dynamics run.

In the first simulation (simulation #1) we simulated the predicted model for 10 nsec to search for the global minimum with defined energy minimized structure. In this simulation, residues within 58 to 291 were hydrogen bond restrained to maintain the secondary structure of the core region. Three cysteine residues present at the position 83,87,90 were distance restrained to maintain the distance between them. N-terminal region (1–57) and C-terminal region (292–361) of the model was allowed to move freely during this simulation. The aim of this simulation was to generate an energetically optimized structure at both N and C terminal regions of the protein. For the second simulation (simulation #2) we took the protein with the energy minimized structure obtained from the first simulation as the starting structure. In this simulation we restrained the position of all the atoms present in defined secondary structure of the protein and the three highly conserved cysteine residues (present at the positions 83, 87 and 90), following which the system was subjected to a 10 nsec molecular dynamics simulation. These atoms were restrained at a fixed reference point by applying force constant in each dimension (x,y,z) having magnitude of 1000 for each atom.

Simulation #2 results in an energetically optimized structure of all the amino acids present in the loop and the coil regions of the protein.

In the third simulation (simulation #3) we took the final optimized protein structure obtained from the previous simulation (simulation #2) as the starting structure. The starting molecule was subjected to a 20 nsec simulation with sulfur atom of the three cysteine residues (at positions 83, 87 and 90) distance restrained with each other as if they were rigidly bounded to the iron atoms of the Fe-S cluster. The changes in the radius of gyration (R_g_) of the wild type Viperin protein was monitored as the simulation progressed.

For the fourth simulation (simulation #4) the cysteine residues at the positions 83, 87 and 90 were mutated with alanine residue using the Discovery studio v2.5.0.9164 program. The mutated protein was subjected to a 20 nsec simulation and the change of R_g_ of the mutated Viperin molecule was monitored with the progress of the simulation. PyMOL (version 0.99) was used as the visualization software.

### Spectroscopic Measurements

Tryptophan fluorescence, UV visible spectra and Circular Dichroism (CD) experiments were performed in 20 mM sodium phosphate buffer (pH 7.5) at room temperature. Absorption spectra were recorded between 200 and 700 nm using a Shimadzu 1700 Pharmaspec UV-VIS spectrophotometer. The absorption peaks at around 325 and 410 nm were used to monitor the Fe-S cluster of the protein.

Steady state fluorescence experiments were performed typically with 1 µM protein samples using a PTI fluorometer (Photon Technology International, USA). The protein samples were excited at 295 nm to avoid any contributions from tyrosine residues and the emission wavelengths were scanned between 315 nm and 415 nm.

CD experiments were carried out using a Jasco J815 spectropolarimeter (Japan Spectroscopic Ltd., Japan). Far UV CD experiments (between 200 nm and 250 nm) were carried out using protein samples of 4–5 uM concentrations. The path length of the cuvette used for the CD measurements was 1 mm. Typically ten spectra were collected in continuous mode and averaged for the CD measurements. The value at 223 nm was plotted against urea concentration to monitor the stability of the WT and cysteine mutants.

Equilibrium unfolding transitions of the WT and cysteine mutants were carried out using protein samples in the presence of different concentrations of urea incubated overnight at room temperature. Far UV CD and steady state tryptophan fluorescence were used to monitor the unfolding transitions of the proteins. The measured data were analyzed assuming a two-state unfolding transition using Equation 1:
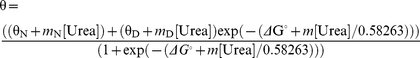
(1)Where θ is the observed spectroscopic change (far UV CD or steady state fluorescence intensity), θ_N_ and θ_D_ are the measured values for the completely folded and completely unfolded proteins extrapolated to zero urea concentration respectively. ΔG° is the free energy of unfolding, and m corresponds to the co-operativity of unfolding transitions.

Data analyses for the spectroscopic measurements were carried out using OriginPro version 7.5 (OriginLab Corporation).

## Results and Discussion

### Computational analyses of the WT and triple cysteine mutant of Viperin

Figure 1(a) explains the domain structure of the full length WT Viperin. Figure 1(b) shows the predicted structure of full length human Viperin generated by a combination of sequence homology modeling and other computational methods. Sequence analysis studies such as data base searching using NCBI protein BLAST [13], multiple sequence alignment performed using MUSCLE [14] and domain search carried out using PRODOM [15] show the presence of an Fe-S motif (CX_3_CX_2_C). It has been shown that these conserved cysteine residues are critical in the anti-viral activity of Viperin [4]. Domain search results show that the protein contains a SAM domain (Figure 1(a)).

An analysis of Viperin sequence using PSI-BLAST [16] indicates the presence of few remote homologs with solved crystal structures. However, the sequence identities between Viperin and these remote homologs are low (for example, MoaA, whose pdb id is 1TV8, has only 23% sequence identity with Viperin). Hence, we employed a strategy consisting of multi-template homology modeling, ab-initio calculations for the loop regions and molecular dynamics simulations for the structure refinement. A detailed flow chart diagram of the used strategy has been shown in Figure S1. Some of the relevant details of the steps used in the used strategy is outlined in the following paragraphs.

Templates for the homology model have been searched by utilizing meta-server program @tome v2.1 [17]. The meta-server program @tome v2.1 searches remote homologues having defined structures with the help of sequence profile search engine PSI-BLAST [16], fold-recognition software such as FUGUE [18] and SP3 [19], profile–profile comparisons based on Hidden Markov-Model through HHSEARCH [20], [21]. The use of ten iterations for each search engine yields twenty biologically significant distant homologs and eighteen of these twenty sequences have CX3CX2C motif. Secondary structure prediction carried out with the help of PSIPRED [22] indicates Viperin as an α-β type protein. It has been shown that the homology model constructed by utilizing multiple templates increases the model quality substantially [23] and hence multiple templates have been selected from the PDB database [24]. For the selection of multiple templates we have utilized [a] sequence profile based on MSA results [b] sequence secondary structure prediction knowledge from PSIPRED and [c] the sequences containing SAM domains. The chosen template proteins include molybdopterin biosynthesis protein A (MoaA) (PDB id- 1TV8, 1TV7, 2FB2, 2FB3); Pyruvate formate-lyase 1-activating enzyme (PDB id- 3C8F); oxygen-independent coproporphyrinogenIII oxidase (PDB id- 1OLT); lysine-2,3-aminomutase from *Clostridium subterminale* SB4 (PDB id- 2A5H); archaeal TYW1, the enzyme catalyzing the second step of wye-base biosynthesis (PDB id-2YX0); and Biotin Synthase (PDB id- 1R30). Figure S2 (Supporting Data) shows the sequence alignment of Viperin and the template proteins used. Homology model of Viperin has been created with the help of Modeller verson9.2 [25] utilizing these chosen template proteins

In the predicted structure, the regions with poor accuracy (the loop regions in particular) are further refined by implementing an ab-initio method using Congen [26]. Congen uses conformational search to explore possible conformation of a loop, and then uses an energy function to rank for the best conformation of the loop. The resulting predicted structure was subjected to a 10 nsec molecular dynamics simulation (hydrogen bond restrained at the core region) to obtain the energy minimized structure of the N and C terminal regions.

The structural regions containing loops and coil have been refined further with the help of position restrained molecular dynamics. Position restraining includes all the atoms present in defined secondary structure of the protein and the three highly conserved cysteine residues (present at the positions 83, 87 and 90). Conformation of un-restrained positions in the protein with lowest potential energy is taken into consideration to completely define Viperin structure. Figure S3 shows a superimposition of the predicted structure of Viperin with a few of the template proteins used.

The acceptability of the final predicted structure has been verified using different methods. First, the structure shows constant RMSD throughout a 20 nsec molecular dynamics simulation (simulation # 3). Thus the final structure can be considered a stable structure. Second, the structure quality has been determined with the help of PROCHECK [27], WHAT IF [28] and PROSA-WEB [29], [30]. Ramachandran plot from the PROCHECK [27] results shows that 98.1% residues of the final structure are present within the allowed region. G-Factors overall average values of −0.37 and −0.20 are obtained for the full-length and the core-region (residues 70–240) structures of Viperin respectively. PROSA-WEB [29], [30] results show that the overall model quality of the full-length protein lies within the crystal structure defined limit for protein with 361 amino acid residues (Z-Score value −4.43). If we consider only the core region of the model, the quality lies within the crystal as well as nmr defined region of protein with same number of amino acids (Z-Score value −4.09). Results from WHAT IF [28] server also show that the packing quality of the predicted structure of the full-length protein is well within the allowed value.

The predicted structure of Viperin (Figure 1(b)) shows a double layer containing both α-helix and β-sheets. Fe-S cluster is present at the bottom of the central pocket within the protein, which is surrounded by parallel beta strands forming the first layer (shown by yellow color in Figure 1(b)). The presence of a second layer of alpha helix is found just above the beta strands (Figure 1(b)). Figure 1(e) shows the position of three cysteine residues (83, 87 and 90) and its amino acid neighborhood in Viperin structure. Fe-S cluster occupies the key central position of Viperin, and is positioned in a core formed by a large number of hydrophobic residues (shown by space-fill model (Figure 1(e)). The hydrophobic interaction is particularly important for Cys83 and Cys87 which are surrounded by hydrophobic residues namely Phe 79, 128; Leu129, 160; Ile154, 161; Val155 for Cys83 and Ile191, 224; Val187, 189, 223, 229; Phe 227 for Cys87. We hypothesized that the removal of the cluster would lead to a large perturbation in the energetic balance of this hydrophobic core that may lead to significant unfolding of the protein.

To test this hypothesis computationally, we have carried out detailed computational analysis to determine the changes in Viperin structure induced by the removal of cysteine residues (simulation # 3 and 4, see materials and method). Predicted structure of the WT Viperin has been chosen as the starting structure in the simulation #3 while the predicted structure with cysteines mutated with alanines is chosen as the starting structure for the simulation # 4. Figure 1(c) shows the variation of radius of gyration (R_g_) with time for the WT Viperin. For the WT protein, R_g_ remains constant with time. The triple mutant, on the other hand, shows a systematic decrease in R_g_ with time (figure 1(d)). These data indicate that for the mutated Viperin protein, the removal of Fe-S cluster from the center of the structure results in the compaction at the central pocket resulting in the collapse of the protein structure.

### Expression and purification of the WT and cysteine mutants of Viperin

**Figure 2:**
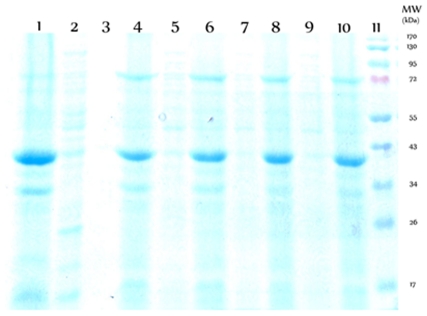
SDS-PAGE electrophoresis analysis of WT and cysteine mutants of Viperin. Sample lanes are as follows: (1) supernatant of WT Viperin (2) inclusion body fraction of WT Viperin (3) supernatant of C90A mutant (4) inclusion body fraction of C90A mutant (5) supernatant of C87A mutant (6) inclusion body fraction of C87A mutant (7) supernatant of C83A mutant (8) inclusion body fraction of C83A mutant (9) supernatant of the triple cysteine mutant (10) inclusion body fraction of the triple cysteine mutant (11) a molecular weight ladder. The WT protein has been found to express well in the supernatant. In contrast, all the cysteine mutants are expressed as inclusion body.

To get detailed insight into some of the above predictions and to understand further the roles of these conserved cysteine residues, we have generated three site directed mutants by replacing each cysteine residue with an alanine residue (C83A, C87A and C90A). Additionally, we have generated a triple mutant where all the three cysteines have been mutated with alanine residues (C83AC87AC90A). Figure 2 shows expression profiles of the WT and cysteine mutants determined by SDS-PAGE gel electrophoresis. The WT protein has been found to express well and as a soluble protein. In contrast, each of the cysteine mutants has been expressed as inclusion body. Inclusion body generally forms because of the formation of hydrophobic aggregates of the partially unfolded or unfolded states, suggesting that the removal of iron-sulfur cluster may lead to destabilization of protein.

### UV-Visible spectroscopy indicates the absence of iron-sulfur cluster in the cysteine mutants

**Figure 3:**
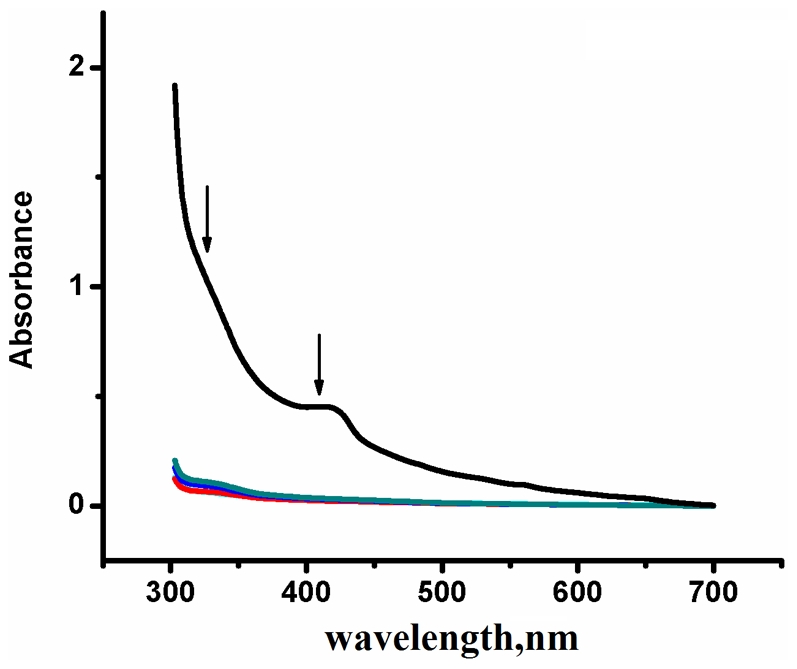
UV-visible absorption spectra of WT and cysteine mutants of Viperin. UV-visible absorption spectra of the WT Viperin (black), the triple cysteine mutant (cyan), C83A mutant (red), C87A mutant (blue), and C90A mutant (dark green). The experiments are performed in 20 mM sodium phosphate buffer at ph7.5. The WT protein exhibits two peaks at 325 nm and 410 nm (shown by arrows) which are characteristic of Fe-S cluster. They are found to be absent in the case of cysteine mutants.

Figure 3 shows the UV–Vis spectrum of the WT Viperin, which exhibits a broad peak at 410 nm which is accompanied by a shoulder at 325 nm. Both peaks disappear upon reducing the protein by adding trace amount of dithionite. The appearance of these characteristic bands indicates the presence of Fe-S cluster as shown before [31].These bands have been observed in the case of recombinant Viperin fragment (45–361) by Shaveta et al. [8] and also in the case of another Viperin fragment (43–340) as reported by Duschene et al. [7]. In sharp contrast, none of the Viperin mutants shows the presence of these absorption bands (325 nm and 410 nm) (Figure 3). The UV-visible absorption results clearly indicate that the replacement of any of the cysteine residues (C83A, C87A or C90A) abrogates the ability of Viperin to bind to iron to form Fe-S clusters. Removal of any one of these cysteine residues removes the Fe-S cluster from the protein. To further confirm, we have prepared two Viperin mutants (R81K) and (Q82N) in which an arginine and a glutamate residues neighboring to the C83 have been replaced. Both these mutants (R81K and Q82N) have been expressed as soluble proteins. Furthermore, the absorption spectra of these two mutants show the presence of Fe-S clusters (Figure S4).

### Circular Dichroism and Steady State Fluorescence suggest that cysteine mutants are conformationally unstable

**Figure 4:**
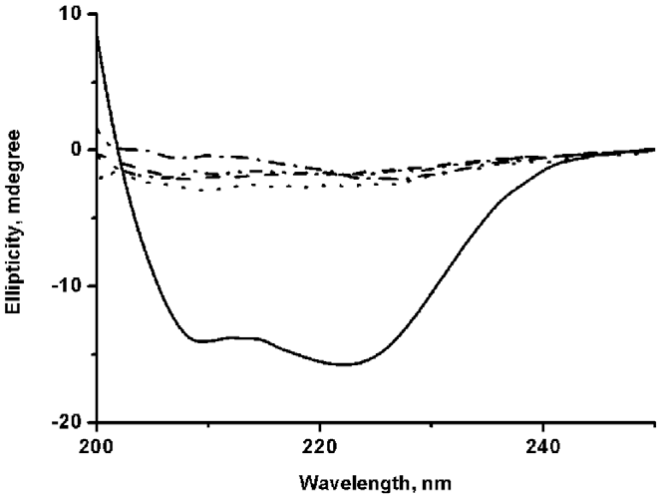
Far-UV circular dichroism spectra of WT and cysteine mutants of Viperin. Far-UV CD spectra of the WT Viperin (____), the triple cysteine mutant (-.-.-.), C83A mutant (_ . . . _), C87A mutant (- - - -), C90A mutant (. . . .). The CD experiments have been carried out in 20 mM sodium phosphate buffer at ph7.5.

Far-UV CD (between 200 and 250 nm) is generally used to monitor the changes in the secondary structure whereas tryptophan fluorescence is used to understand the changes in the local tryptophan regions of a protein. Near UV CD, which is normally used to monitor the tertiary structure of a protein, could not be used in our study since this technique requires high concentration and the cysteine mutants show aggregation related complications. Figure 4 shows far UV CD of the WT and cysteine mutants of Viperin. Far UV CD of the WT protein is characterized by the presence of two peaks at 208 nm and 223 nm suggesting the presence of large alpha helical content in its secondary structure. This observation is in agreement with our predicted model (Figure 1(b)). In contrast, a large decrease in the helical content has been observed for each of the cysteine mutants (Figure 4). A comparison between the far UV CD spectra of the WT and the mutant proteins in the folded and unfolded state (in the presence of 10 M urea) shows approximately 88%, 88%, 83% and 85.5% decrease in the helical content in the case of the triple mutant, C83A, C87A and C90A mutants respectively. The triple mutant (C83AC87AC90A) and C83A mutant of Viperin has been found to contain the least extent of secondary structures.

**Figure 5:**
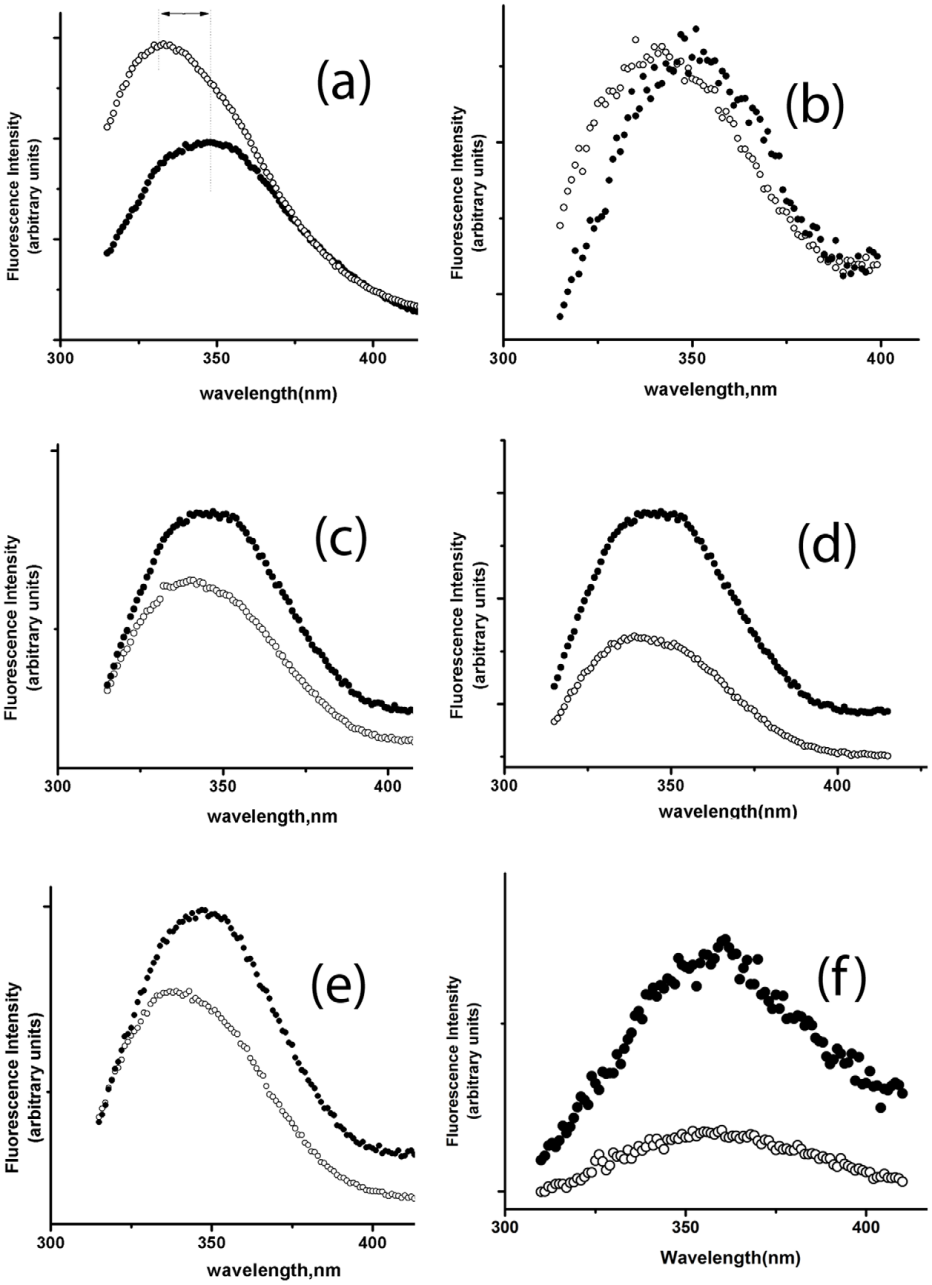
Fluorescence spectra of WT and cysteine mutants of Viperin in folded and unfolded condition. Fluorescence emission spectra of (a) the WT Viperin, (b) the triple mutant (c) C83A mutant, (d) C87A mutant and (e) C90A mutant (f) NATA in the absence (void circle) and presence (closed circle) of 10 M urea. Fluorescence experiments have been carried out in 20 mm phosphate buffer at pH 7.5. A red shift in the emission spectra is shown by a double headed arrow for the WT protein.

Figure 5 shows the fluorescence spectra of the WT and mutant proteins in their folded and unfolded states (in the presence of 10 M urea). The steady state fluorescence spectrum of the WT protein (Figure 5(a)) shows the presence of the emission maximum at 332 nm. This is significantly red-shifted compared to the emission maxima observed for the native states of azurin (308 nm), myoglobin (321 nm) or parvalbumin (316 nm), but significantly blue-shifted compared to the native state of choleratoxin (340 nm) [32]. For azurin, myoglobin or parvalbumin, the tryptophan residue (s) have been shown buried completely within the hydrophobic core of the protein without any water exposure. For cholera-toxin and other similar proteins, tryptophan residue(s) are exposed. The emission maximum of 332 nm observed for Viperin suggests the possibility of the presence of two classes of tryptophans as suggested by Vivian and Callis [30]. The tryptophan residues belonging to the first class reside within the hydrophobic core while the other class is relatively solvent exposed. A careful observation of the predicted structure of Viperin (Figure 1(b)) indicates Tryptophan residues 34 and 165 are buried well inside the hydrophobic core, tryptophan residues 25, 245, and 361 are partially exposed while tryptophans 40, 210 and 352 are completely exposed. Upon unfolding by 10 M urea, the emission maximum of the WT protein shifts to 349 nm as the tryptophan residues become exposed to water. A large decrease in the fluorescence intensity of the WT protein is also observed in the presence of 10 M urea (Figure 5(a)). This happens because of the quenching of the tryptophan residues by aqueous medium as the protein becomes solvent exposed as a result of the unfolding. The fluorescence spectra of cysteine mutants in their native state yield an emission maximum at around 342 nm which is considerably red shifted compared to the WT spectrum (Figure 5(b, c, d, e)). Moreover, the large decrease in fluorescence intensity observed for the unfolding of the WT protein has been found absent for the mutants (Figure 5(b, c, d, e)). Instead the fluorescence intensity of the mutant protein increases in the presence of urea (with the exception of the triple mutant where all the three cysteine residues are replaced, Figure 5(b)). To understand the urea induced increase in the fluorescence intensity we have studied N-acetyl tryptophanamide (NATA) in the absence and presence of 10 M urea. NATA is an excellent representative of a tryptophan residue which is completely exposed. The fluorescence intensity of NATA has been found to increase with the addition of urea (Figure 5(f)) which is similar to the behavior of the cysteine mutants (Figure 5(c, d, e)). These observations clearly suggest that the tryptophan residues in the cysteine mutant proteins are exposed to the aqueous environment even in the absence of urea. Structural analyses using steady state fluorescence and far UV CD thus indicate that the presence of Fe-S cluster play a valuable role in the correct folding of Viperin. The absence of Fe-S cluster results in a large decrease in the secondary and tertiary structure of the protein.

**Figure 6:**
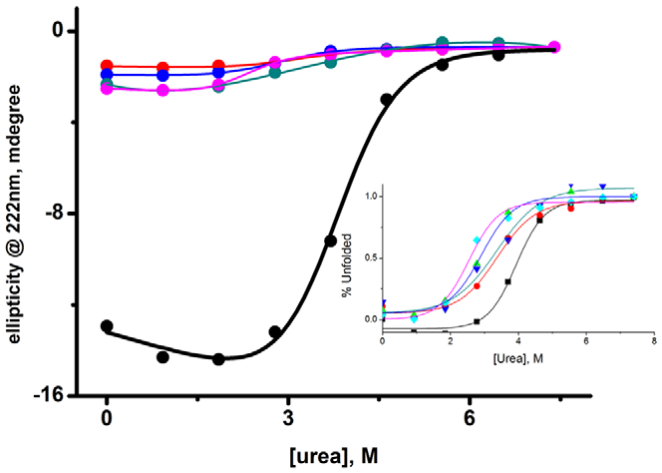
Unfolding transitions of WT and cysteine mutants of Viperin monitored by far- UV CD. Urea induced unfolding of the WT (black), the triple mutant (red), C83A mutant (blue), C87A mutant (dark green), and C90A mutant (magenta) of Viperin. The ellipticity measured at 223 nm is plotted against urea concentration. The data for WT and the cysteine mutants are fit to equation 1 assuming two state unfolding transitions. The experiments have been carried out in 20 mM phosphate buffer at pH 7.5.

To understand the role Fe-S cluster plays in Viperin folding, far-UV CD spectroscopy is used to monitor unfolding transitions of the WT and cysteine mutants. The decrease in CD intensity at 223 nm with urea concentration corresponds to the unfolding of the secondary structure of an alpha-helical protein. Figure 6 shows the variation of mean residue ellipticity of the WT and mutant proteins with urea concentration. The transitions are fit to a model assuming two-state unfolding transitions (equation 1) and the parameters obtained are listed in Table 1. As shown in Table 1, the free energy of unfolding (ΔG°) and the co-operativity parameter (m) of the WT protein are significantly higher compared to the cysteine mutants.

**Table 1:** Thermodynamic parameters of the equilibrium unfolding transitions of WT and cysteine mutants of Viperin.

	ΔG_0_, kcalmol^−1^	m, kcalmol^−1^M^−1^	Mid-point, M
WT Viperin	6.3	1.65	3.82
Triple Mutant	3.25	1.1	2.95
C83A	3.25	1.1	2.95
C87A	3.3	1.2	2.75
C90A	3.5	1.5	2.33

Equilibrium unfolding transitions of the WT and cysteine mutants have been monitored also by steady state fluorescence spectroscopy. Tryptophan fluorescence is a convenient probe to monitor the unfolding of the local tryptophan environment. As observed by far UV CD, the equilibrium unfolding transition of the WT Viperin monitored by steady state fluorescence can be fit to a typical two state transition model (results not shown). In contrast, the fluorescence data do not show any distinct transition for any of the cysteine mutants. This is expected because the tryptophan residues are found to be solvent exposed in the native states of the cysteine mutants (as shown before in Figure 5) as a result of the partial unfolding. Consequently, the addition of urea does not change the environment of the tryptophan residues.

**Figure 7:**
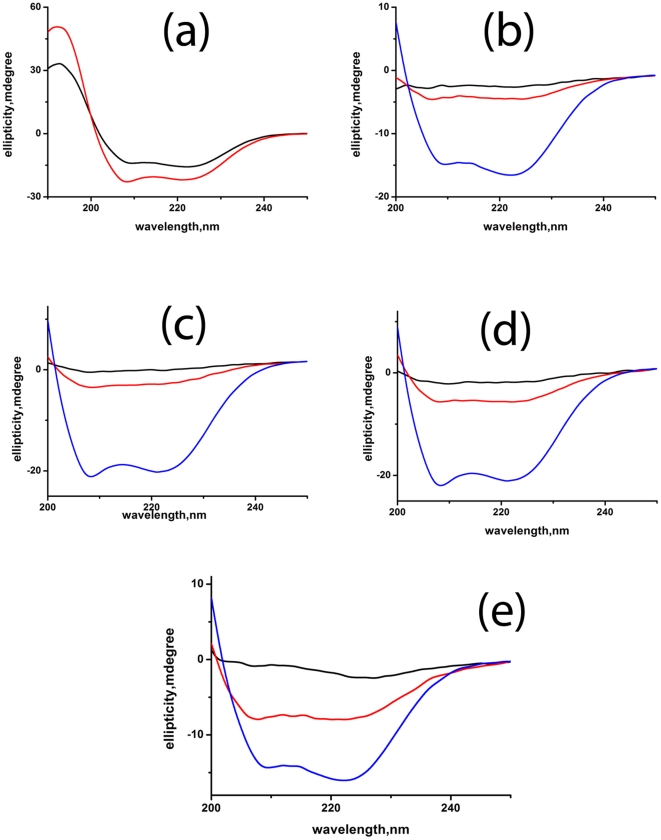
Effect of TFE on the structure of WT and cysteine mutants of Viperin monitored by Far-UV CD. Far UV-CD spectra of (a) the WT (b) the triple cysteine mutant (c) C83A mutant (d) C87A mutant and (e), C90A mutant of Viperin in the presence (red) and absence of 50% TFE (black). Far UV-CD spectrum of WT Viperin (blue) has also been shown (7(b)-7(e)). All these experiments have been carried out in 20 mM sodium phosphate buffer at pH 7.5.

To determine whether this loss of helical content of the cysteine mutants can be reverted by the addition of a helix forming reagent, we have studied the effect of trifluoroethanol (TFE) on the WT and mutant proteins. TFE has been shown to induce alpha helix and also to stabilize alpha helical proteins and their fragments [33]–[36]. The effect of TFE on the secondary structure of WT and cysteine mutant proteins is shown in the figure 7.

The addition of TFE leads to an increase in the percentage of α-helix for all the proteins as observed by the increase in the ellipticity although the extent of increase varies. The percentage of increase for the mutant proteins has been calculated from the difference in the ellipticity of the protein in the absence and presence of 50% TFE and assuming that the ellipticity of the WT protein to be 100%. The percentage of increase for the C90A mutant is maximum and almost 46% of secondary structure is retained after the addition of 50% TFE (Figure 7(e)). The percentage of refolding is minimum (13%) in the case of triple mutant (Figure 7 (b)). The C83A and C87A mutants show 19% (Figure 7(c)) and 27% (Figure 7(d)) of increase respectively.

Metal cofactors play diverse roles to maintain the structural and conformational integrity of a protein. For example, the removal of iron containing co-factors, heme, from myoglobin, horseradish peroxidase or lactoperoxidase shows only a minimal effect [37]. Heme removal from cytochrome c, on the other hand, leads to large change in the secondary and tertiary structures [38]. While the apo-protein of Cu-Zn superoxide dismutase retains majority of the secondary and tertiary structure of the holo protein [39], loss of iron-sulfur clusters from biotin synthase leads to protein degradation [40]. Apo and holo forms of zinc containing adenosine deaminase are shown to have limited variability in there molecular structures although a significant difference in the stability has been observed [41].

In this paper we use a combination of multiple template homology modeling and ab-initio calculations to obtain a predicted structure of Viperin to be a α-helix β-sheet protein containing a Fe-S cluster which holds the double-layered structure of the protein. Molecular dynamics simulation shows that the removal of the cluster would lead to instability resulting in the collapsing of the tertiary structure of the protein. We also show that the replacement of any or all of these three cysteine residues (83, 87 and 90) leads to complete absence of 325 nm and 410 nm absorption bands. Since these two bands represent the presence of the Fe-S cluster in the protein, this result implies that the presence of each of these cysteine residues (83, 87, and 90) is needed for the presence of Fe-S cluster. The far UV CD of WT Viperin shows two significant negative peaks at 223 nm and 209 nm, which suggests Viperin to be a predominately α-helical protein. Analysis of the far UV CD data estimates the presence of about 60% α-helix and 40% β-sheet in the structure of the WT protein. The removal of the Fe-S cluster in the cysteine mutants lead to a large decrease in the secondary structure of the protein. However, they are not completely unfolded since far UV CD of all the mutant proteins show unfolding of some residual structures by urea. On the other hand, unfolding of the cysteine mutants monitored by steady state fluorescence spectroscopy does not show any transition. These results along with the fact that these mutants show fluorescence emission maxima identical to those of the unfolded proteins suggest that the aromatic regions of these mutants are completely unfolded even in the absence of urea although there could be residual secondary structure present in the cysteine mutants. All the three mutants show significantly less stability as shown by a large decrease in the free energy of unfolding (ΔG_0_ of the mutant proteins are about 3.5 kcalmol^−1^ compared to that of 6.3 kcalmol^−1^ for the WT protein, Table 1). In addition, the mutant proteins are aggregation prone as observed by their expression in the inclusion body while WT Viperin is expressed well as a soluble protein.

The mechanism of Viperin's antiviral effect is not understood. It is also not clear whether Viperin as a SAM enzyme has any mechanistic role to play in its ability to interact with farnesyldiphosphatesynthetase (FPPS). It is not known whether the presence of metal cofactor has any effect to disrupt the formation of lipid rafts and consequent increase in the mobility of the plasma membrane (2, 5). It has been shown earlier that the triple mutant described in this paper does not show antiviral activity. The present results show severe lack of conformational integrity of the triple mutant of Viperin resulting its self-association. For other proteins like apolipoproteins and synucleins self-association has been shown to interfere directly with the membrane association [42]. It has been shown recently that apolipoprotein E interacts with the membrane only in its monomeric form and does not while it is self-associated [42]. Self-associated Viperin mutants may have similar effects towards its interaction with membrane.

## Supporting Information

Figure S1 **A flow chart of the strategy used for the development of the structural model of Viperin.** A simple homology search (pathway 1) did not find any close homolog with solved structure. Remote homology search leads to pathway 2 and 3. A combination of multi-template homology modeling, ab-initio type calculations and molecular dynamic simulations (pathway 3) results in the final predicted structure. C_α_ RMSD values are obtained by utilizing DaliLite program (Holm L, Park J (2000) DaliLite workbench for protein structure comparison. Bioinformatics (Oxford, England). 16 (6): 566-7).
(TIF)

Figure S2 **Sequence alignment of Viperin with nine different template proteins.** The figure has been prepared using Jalview (Waterhouse AM, Procter JB, Martin DMA, Clamp M, Barton GJ (2009) Jalview Version 2—a multiple sequence alignment editor and analysis workbench. Bioinformatics 25: 1189–1191.) with ClustalX (Thompson JD, Gibson TJ, Plewniak F, Jeanmougin F, Higgins DG (1997) The CLUSTAL X windows interface: flexible strategies for multiple sequence alignment aided by quality analysis tools. Nucleic Acids Res 25: 4876–4882.) coloring option.
(TIF)

Figure S3 **A superimposition of the predicted structure of Viperin with five representative template proteins.** Viperin is colored green and five other template structures, namely 1TV8,1TV7,2FB2,2FB3,3C8F are shown using cyan, yellow, magenta, light pink and white colors respectively.
(TIF)

Figure S4 **Absorption spectrum of R81K mutant of Viperin.** Absorption bands at positions 325 nm and 410 nm show the presence of Fe-S cluster. Absorption spectrum of Q82N is identical and not shown.
(TIF)
