# Supplementary figures and images for: The Presence of the Iron-Sulfur Motif Is Important for the Conformational Stability of the Antiviral Protein, Viperin
Source: PLoS One. 2012 Feb 21;7(2):e31797. doi: 10.1371/journal.pone.0031797 (PMC3283665; doi:10.1371/journal.pone.0031797)

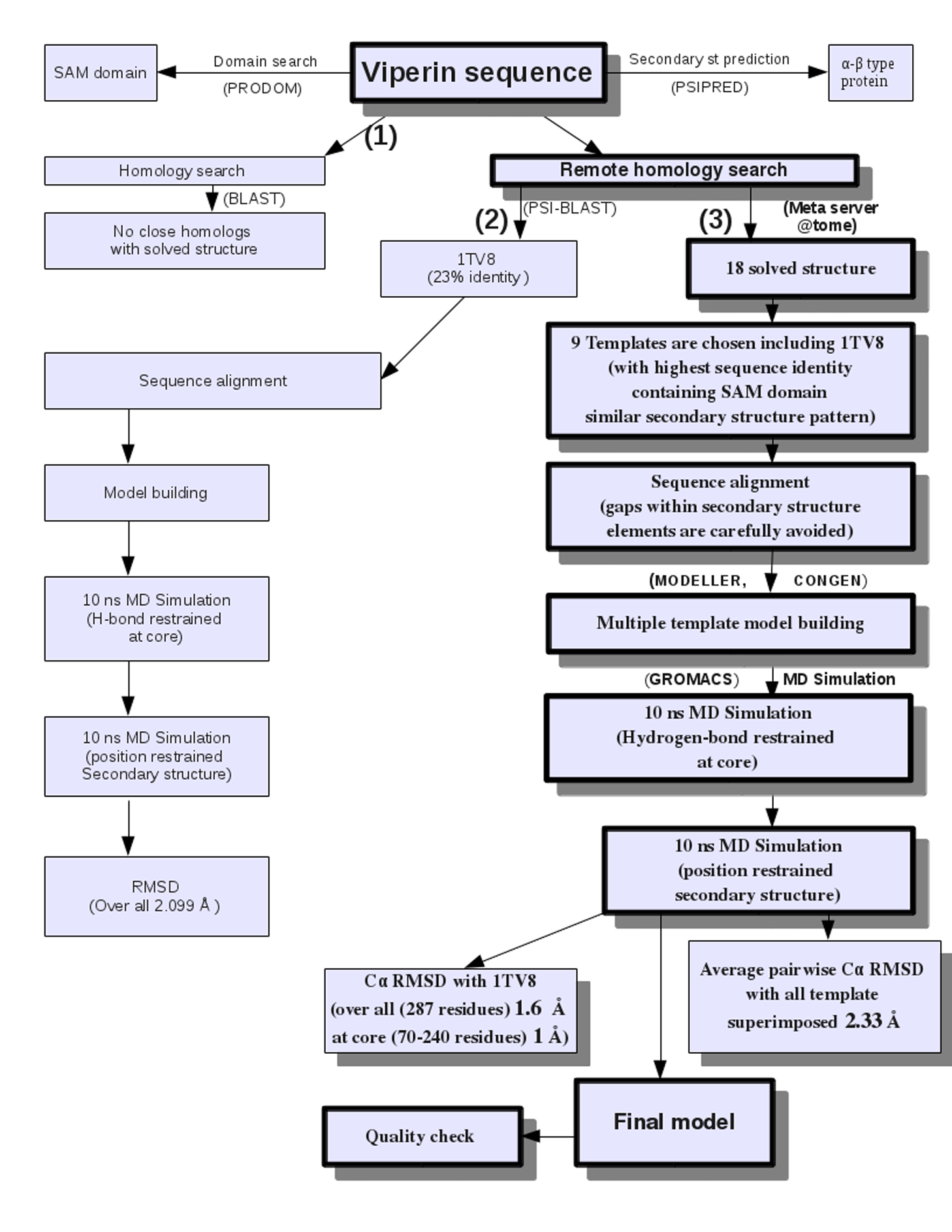

Supplement: Figure S1 — A flow chart of the strategy used for the development of the structural model of Viperin. A simple homology search (pathway 1) did not find any close homolog with solved structure. Remote homology search leads to pathway 2 and 3. A combination of multi-template homology modeling, ab-initio type calculations and molecular dynamic simulations (pathway 3) results in the final predicted structure. Cα RMSD values are obtained by utilizing DaliLite program (Holm L, Park J (2000) DaliLite workbench for protein structure comparison. Bioinformatics (Oxford, England). 16 (6): 566-7). (TIF) [file pone.0031797.s001.tif]

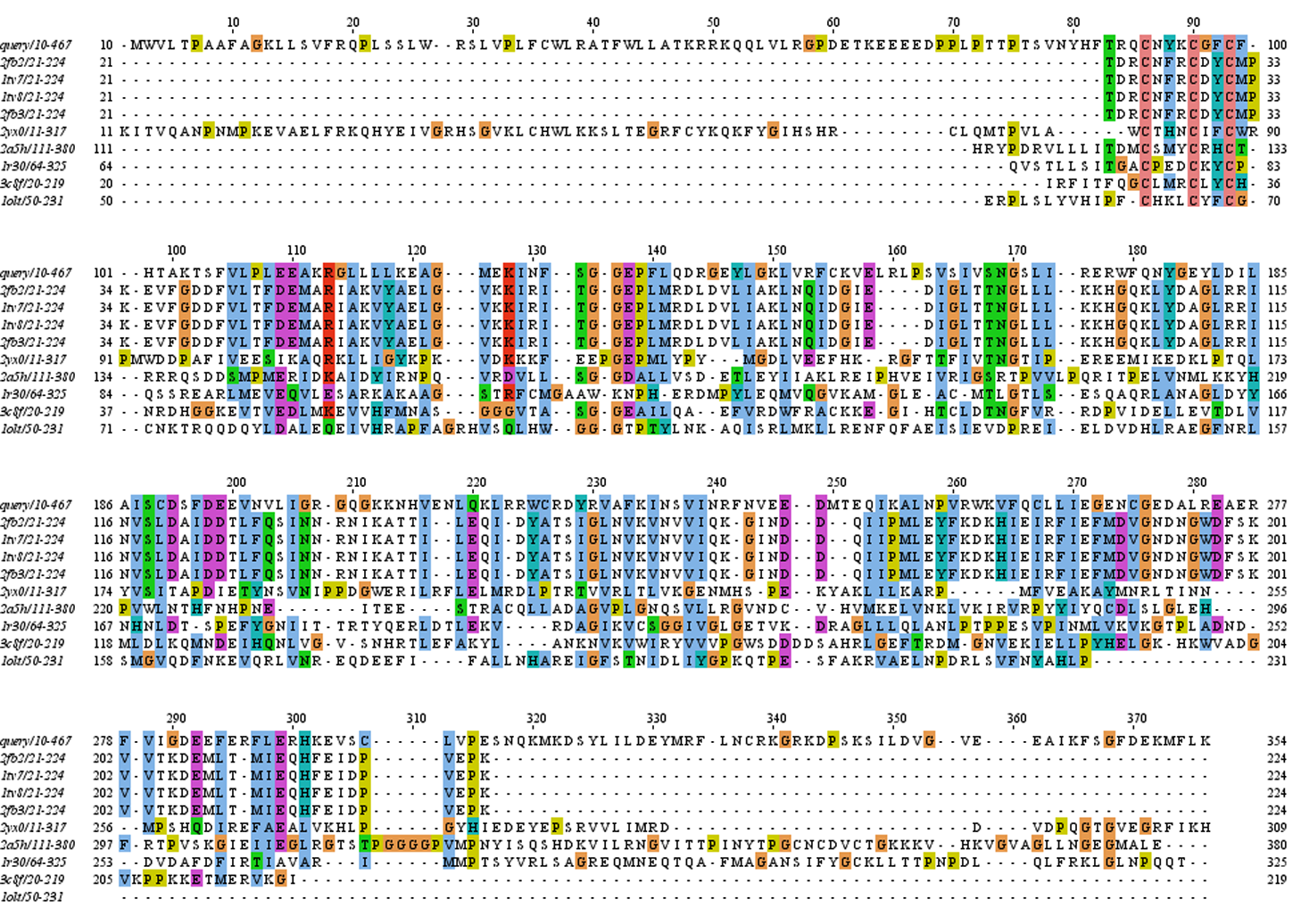

Supplement: Figure S2 — Sequence alignment of Viperin with nine different template proteins. The figure has been prepared using Jalview (Waterhouse AM, Procter JB, Martin DMA, Clamp M, Barton GJ (2009) Jalview Version 2—a multiple sequence alignment editor and analysis workbench. Bioinformatics 25: 1189–1191.) with ClustalX (Thompson JD, Gibson TJ, Plewniak F, Jeanmougin F, Higgins DG (1997) The CLUSTAL X windows interface: flexible strategies for multiple sequence alignment aided by quality analysis tools. Nucleic Acids Res 25: 4876–4882.) coloring option. (TIF) [file pone.0031797.s002.tif]

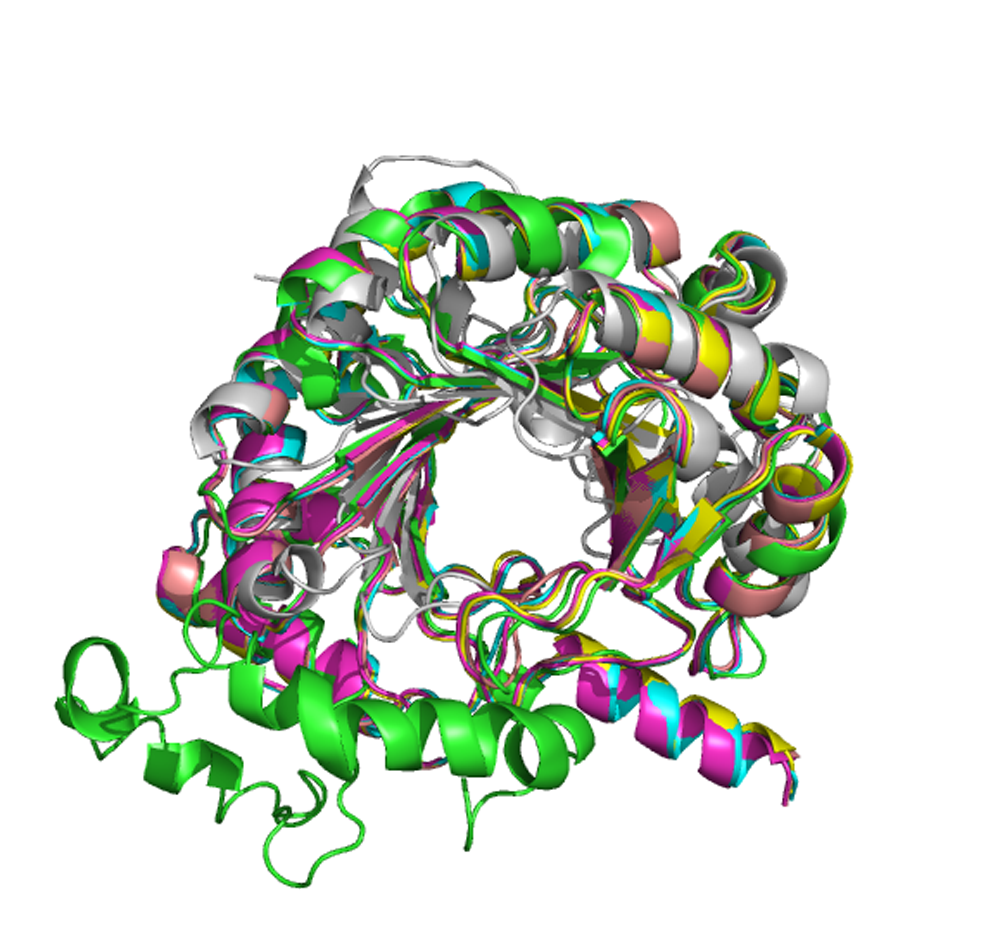

Supplement: Figure S3 — A superimposition of the predicted structure of Viperin with five representative template proteins. Viperin is colored green and five other template structures, namely 1TV8,1TV7,2FB2,2FB3,3C8F are shown using cyan, yellow, magenta, light pink and white colors respectively. (TIF) [file pone.0031797.s003.tif]

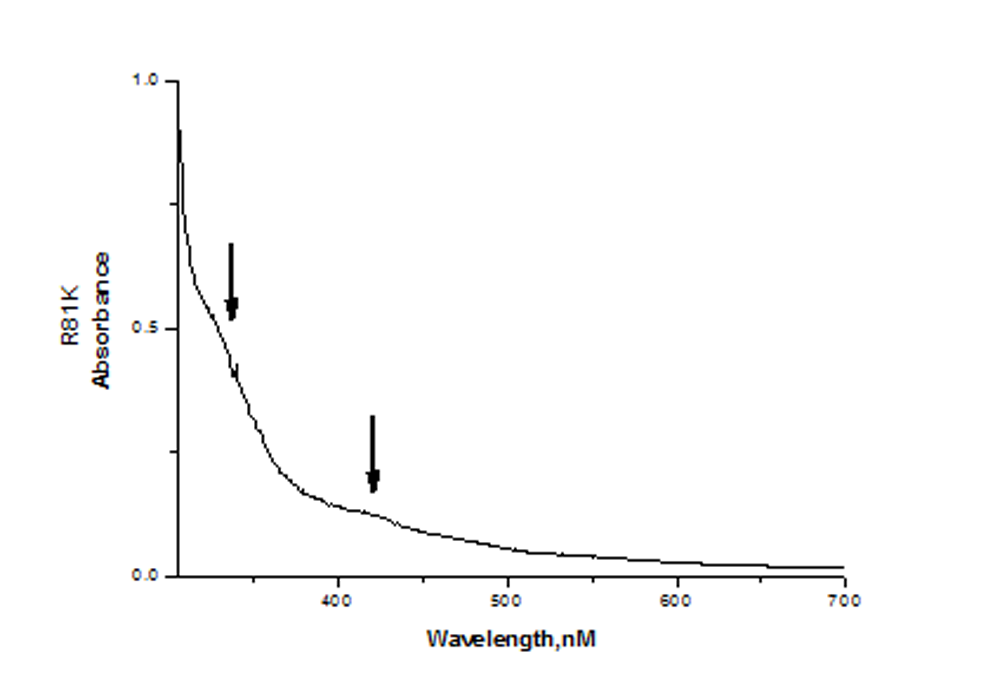

Supplement: Figure S4 — Absorption spectrum of R81K mutant of Viperin. Absorption bands at positions 325 nm and 410 nm show the presence of Fe-S cluster. Absorption spectrum of Q82N is identical and not shown. (TIF) [file pone.0031797.s004.tif]
